# Evaluation of Glycated Hemoglobin (HbA1c) for Diagnosing Type 2 Diabetes and Prediabetes among Palestinian Arab Population

**DOI:** 10.1371/journal.pone.0088123

**Published:** 2014-02-05

**Authors:** Akram T. Kharroubi, Hisham M. Darwish, Ahmad I. Abu Al-Halaweh, Umaiyeh M. Khammash

**Affiliations:** 1 Department of Medical Laboratory Sciences, Faculty of Health Professions, Al-Quds University, Jerusalem, Palestine; 2 Faculty of Medicine, Molecular Genetics Laboratory, Al Quds University, Jerusalem, Palestine; 3 Augusta Victoria Hospital, Jerusalem, Palestine; 4 United Nation Relief and Working Agency (UNRWA), Jerusalem, Palestine; University of Tolima, Colombia

## Abstract

The purpose of the study is to compare the potential of HbA1c to diagnose diabetes among Palestinian Arabs compared to fasting plasma glucose (FPG). A cross-sectional sample of 1370 Palestinian men (468) and women (902) without known diabetes and above the age of 30 years were recruited. Whole blood was used to estimate HbA_1c_ and plasma for FPG and total lipid profile. Fasting plasma glucose was used as a reference to diagnose diabetes (≥ 126 mg/dL) and prediabetes (100–125 mg/dL). The area under the receiver operating characteristic curve (AUC) for HbA_1c_ was 81.9% to diagnose diabetes and 63.9% for prediabetes. The agreement between HbA_1c_ and diabetes as diagnosed by FPG was moderate (ĸ  =  0.498) and low with prediabetes (ĸ = 0.142). The optimal cut-off value for HbA1c to diagnose diabetes was ≥ 6.3% (45 mmol/mol). The sensitivity, specificity and the discriminant ability were 65.6% (53.1–76.3%), 94.5% (93.1–95.6%), 80.0% (72.8–87.3%), respectively. However, using cut-off value of ≥ 6.5% (48 mmol/mol) improved specificity. At this cut-off value, the sensitivity, specificity and the discriminant ability were 57.4% (44.9–69.0%), 97.1% (96.0–97.9%) and 77.3% (71.0–83.5%). For diagnosing prediabetes with HbA1c between 5.7–6.4% (39–46 mmol/mol), the sensitivity, specificity and the discriminant ability were 62.7% (57.1–67.9%), 56.3% (53.1–59.4%) and 59.5% (56.3–62.5%), respectively. HbA_1c_ at cut-off value of ≥ 6.5% (48 mmol/mol) by itself diagnosed 5.3% and 48.3% as having diabetes and prediabetes compared to 4.5% and 24.2% using FPG, respectively. Mean HbA_1c_ and FPG increase significantly with increasing body mass index. In conclusion, the ROC curves showed HbA1c could be used for diagnosing diabetes when compared to FPG but not for prediabetes in Palestinians Arabs even though only about 50% of the diabetic subjects were identified by the both HbA1c and FPG.

## Introduction

The Center for Disease Control (CDC) reported a world-wide prevalence of diabetes in its national diabetes fact sheet to be 11.57% [1]. According to Hare et al. [2], diabetes mellitus is the greatest public threat of the 21^st^ Century with currently 285 million people world-side having diabetes and is expected to double to 439 million by 2030 with an additional half billion people are expected to be at high risk. These are conservative figures since, on one hand, type 2 diabetes mellitus is spreading among the young generation due to changes in their life style all over the world and, on the other hand, new diagnostic criteria of diabetes mellitus using HbA1c is emphasizing specificity over sensitivity as recommended by the International Expert Committee [3] which may underestimates the prevalence of diabetes [4].

Since the recommendation of the International Expert Committee in 2009 to use HbA_1c_ test to diagnose diabetes [3], the American Diabetes Association (ADA), the Endocrine Society [5], the Word Health Organization [6] and most scientists in different countries all over the world have endorsed using HbA_1c_ to diagnose diabetes. The advantages of using HbA_1c_ over fasting plasma glucose (FPG) to diagnose diabetes include greater convenience and preanalytical stability, lower CV (3.6%) compared to FPG (5.7%) and 2h – Oral Glucose Tolerance Test (OGTT) (16.6%). Stronger correlation with microvascular complications especially retinopathy, a marker for glycemic control and glycation of proteins is the direct link between diagnosis of diabetes and its complications [7]–[12].

Most studies with different ethnic groups have endorsed a cut-off value for an HbA_1c_ of ≥ 6.5% (48 mmol/mol) to diagnose diabetes as has been recommended by the International Expert Committee [3]. The ADA considers people to be at high risk (prediabetes) if HbA_1c_ is 5.7–6.4% (39–46 mmol/mol) [4]. Different cut-off values have been reported for diagnosing diabetes in different ethnic groups and ethnicity seems to have a strong influence on cut-off values to diagnose diabetes [13], [14]. Cut-off values of 5.5% (37 mmol/mol) [15]; 6.5% (48 mmol/mol) [16] have been reported in a Japanese studies, 6.0% (42 mmol/mol) in National Health and Nutrition Examination Survey (NHANES III), 6.2% (44 mmol/mol) in a Pima Indian study, 6.3% (45 mmol/mol) in an Egyptian study as reported by Davidson [8]; and three cut-off values for Chinese [14]. The Australians recommended the use of two cut-off values; ≤ 5.5% to ‘rule-out’ and ≥ 7.0% to ‘rule-in’ diabetes [17]. Variations in prevalence of diabetes [18]–[22] and prediabetes [23] due to ethnicity have been documented.

Evaluating the cut-off value for diagnosing diabetes using HbA1c in Arabs needs to be investigated. One report investigated adult Arabs living in Detroit, USA [24] and another one from Abu Dhabi, United Arab Emirates [25]. This is the first report on using HbA_1c_ to diagnose diabetes in adult Palestinians living in, and in the neighborhood of, the refugee camps in the center and southern locations of the West Bank region in Palestine.

## Materials and Methods

### Ethics Statement

Ethical approval for the study protocol was obtained from the Research Ethics Committee of Al-Quds University in the Palestine. Written informed consent was obtained from all participants involved in the study.

### Participants

A convenient cross-sectional sample of 1370 adult Palestinian men (468) and women (902) without known diabetes and above the age of 30 years were recruited (based on their volunteer attendance to clinics) from central and southern refugee camps in Ramallah, Bethlehem and Hebron districts administered by UNRWA. All subjected were instructed to fast for 10–12 hours before coming to the clinic at 8:00 am. A special questionnaire concerning family history and health-related information was filled for all participants by direct interviews with the researchers. People previously diagnosed with diabetes or hemoglobinopathies were ruled out from the study. Blood samples were collected from all subjects using EDTA tubes and centrally analyzed for HbA_1c_. Plasma was also used to analyze FPG and total lipid profile, total cholesterol (TC), triglycerides (TG), high-density lipoprotein (HDL) and low-density lipoprotein (LDL). Blood pressure (BP) and BMI were also measured by the medical staff in the clinics. Body mass index (BMI) in kg/m^2^ was categorized as normal (BMI < 25), overweight (BMI ≥ 25 to < 30) and obese (BMI ≥ 30). The cut-off values for diabetes using FPG was ≥ 126 mg/dL, prediabetes 100–125 mg/dL and normal subjects having FPG < 100 mg/dL. Specificity, sensitivity, and the area under the ROC curve (AUC) for HbA_1c_ using different cut-off values were calculated using FPG as the “gold standard”.

### Analytical procedures

Blood samples were tested for FPG, HbA_1c_, total lipid profile including TC, TG, LDL, and HDL. Fasting plasma glucose and total lipid profile (TC, TG, HDL) were measured enzymatically using Chemwell chemistry analyzer (Awareness Tech, USA), LDL- cholesterol (C) was calculated from the equation of Friedewald equation (LDL–C  =  TC – [HDL–C + (TG/5)]). HbA_1c_ was measured using 3 µL EDTA blood by ion-exchange HPLC using TOSOH G8. Hemoglobin levels and CBC were measured for anemia evaluation as well hemoglobin variants were analyzed because of their interference with HbA_1c_ levels. HbA_1c_ assay was standardized to the Diabetes Control and Complications Trial (DCCT) assay method.

### Statistical analysis

Statistical analysis was performed using SPSS 17.0 software (SPSS Inc., Chicago, IL, USA). Pearson’s correlation coefficient was used to test for co-linearity between the continuous variables, statistical comparisons between different groups for these continuous variables were carried out using Student’s t test and ANOVA. Pearson’s Chi-Square statistic was used to assess for relationships between categorical variables. Receiver operating characteristic (ROC) curves were constructed to calculate sensitivity, specificity, predictive value positive (PVP) and predictive value negative (PVN) at different cut-off values for HbA_1c_. Kappa (ĸ) coefficients were used to test for agreement between HbA_1c_ and diabetes status based on FPG levels (diabetes and prediabetes). The ROC curve plots the sensitivity against 1 minus the specificity at all possible HbA1c cut-off values. The higher the AUC, the better the predictive value of HbA1c based on the logistic regression model. An AUC value of 0.5 would indicate no predictive value, whereas a value of 1.0 would indicate perfect predictive value with no false positives or false negatives. Sensitivity at each possible HbA1c cut-off value was calculated as [TP/(TP +FN)] X 100, where TP  =  true positive (diabetic FPG and HbA1c cut-off value) and FN  =  false negative (diabetic FPG, ≤ cut-off value for HbA1c). The sensitivity represents the percentage of those with fasting plasma glucose < 126 mg/dL (7.0 mmol/L) who are classified as positive according to HbA1c. Specificity was calculated as [TN/(TN + FP)] X 100, where TN  =  true negative (non-diabetic FPG and ≤ cut-off value for HbA1c) and FP  =  false positive (nondiabetic FPG, > cut-off value for HbA1c). The specificity represents and percentage of those with FPG < 126 mg/dL (7.0 mmol/L) who are classified as negative according to the HbA1c. Youdin index and the discriminant ability at each cut-off value for HbA1c were used to determine the optimal cut-off value for HbA1c to diagnose diabetes. The discriminant ability is the average of sensitivity and specificity at each cut-off value. Venn diagrams were used for visual display or concordance/ discordance between FPG and HbA1c-based classification of participants. Statistical significance was accepted at p<0.05. Because of missing values the number of each group in different comparisons is different.

## Results

Fifty percent of the subjects that participated in this study were between the age of 40–49 years. They had no previous diagnosis of diabetes, but 56% of them had a family history of diabetes. The percentage of subjects with hypertension defined as systolic BP ≥ 120 mmHg or diastolic BP ≥ 90 mmHg was about 5.1 and 6.1, respectively. The percentage of subjects with High TC (≥ 5.5 mmol/L), TG (≥ 2.0 mmol/L) and LDL (≥ 3.5 mmol/L) was 16.2%, 15.4% and 27.1% respectively; whereas the percentage of subjects with high HDL (≥ 2.0 mmol/L) were 36.8%. The mean values of age, FPG, HbA_1c_, TC, LDL were not significantly different between males and females whereas mean values of systolic BP, diastolic BP, and TG were significantly higher (p<0.001) in males compared to females and mean HDL and BMI were significantly higher (p<0.001) in females compared to males using t test to compare means.

**Figure 1:**
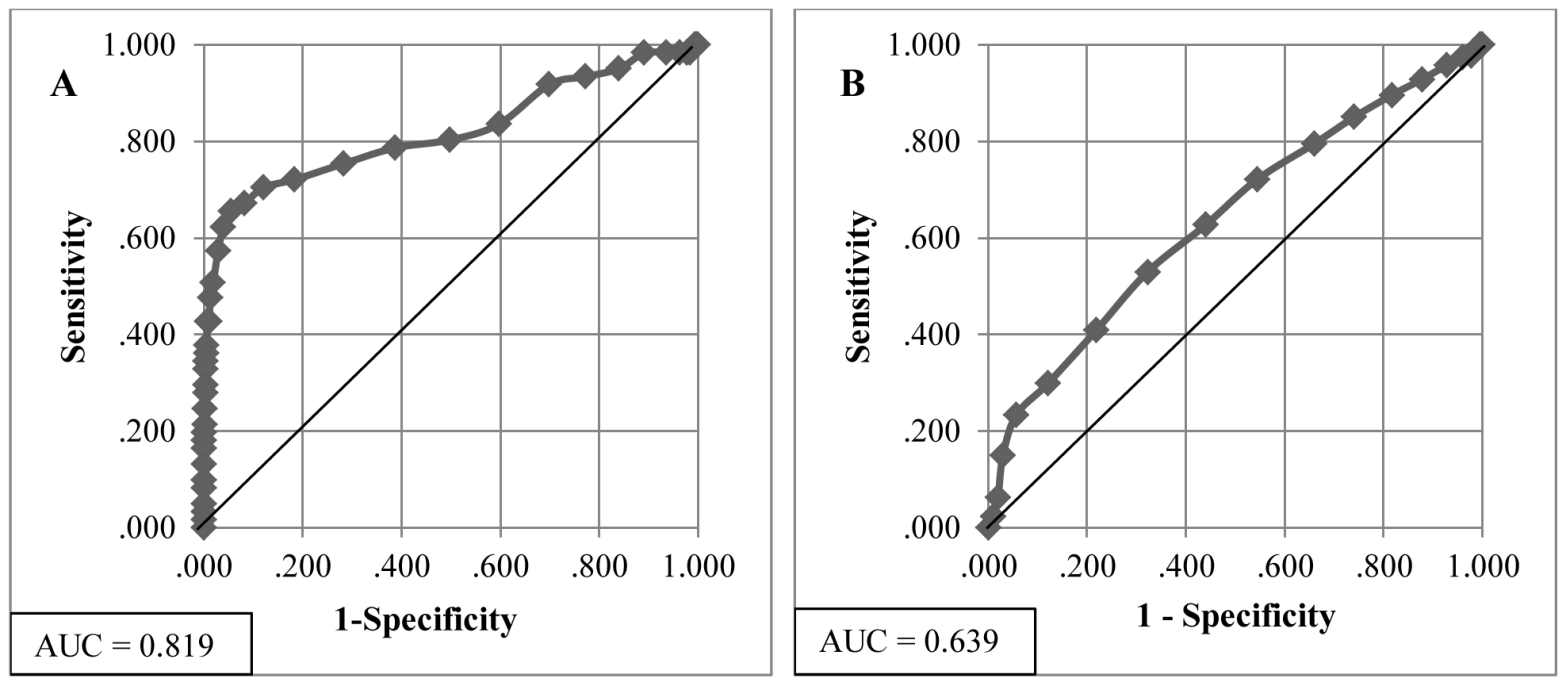
HbA_1c_ receiver operating characteristic (ROC) curves for diabetes (A) and prediabetes (B) using FPG as a reference. AUC: area under the receiver operating characteristic curve.

Figure 1 shows the ROC curves for HbA_1c_ using FPG as a reference. The area under the ROC curve is 0.819 (0.75–0.89) for diagnosing diabetes (Figure 1 A) and 0.639 (0.60–0.68) for prediabetes (Figure 1 B). The agreement between HbA1c and diabetes was moderate (ĸ = 0.498) and low with prediabetes (ĸ = 0.142). The cut-off value of equal sensitivity and specificity or the closest point to 100% sensitivity for diagnosing diabetes was about 5.9% (41 mmol/mol).

Different cut-off values were tested for their ability to diagnose diabetes using FPG as the gold standard. Table 1 shows that HbA_1c_ with cut-off value of ≥ 6.3% (45 mmol/mol) has the highest discriminant ability (80.0%) with sensitivity of 65.6% and specificity of 94.5%, Youden index also gave an optimum cut-off value of ≥6.3. A cut-off value of ≥ 6.5% (48 mmol/mol) gave a specificity of 97.1% and a reasonable sensitivity (57.4%). However, a cut-off value of ≥ 5.9% (41mmol/mol) gave a specificity of 71.1% and a sensitivity of 75.4%. Lower cut-off values less than 5.9% (41mmol/mol) gave poor specificity. The percentage of subjects diagnosed as having diabetes using FPG (≥ 126 mg/dL) and HbA_1c_ at cut-off values of 6.5% (48 mmol/mol), 6.3% (45 mmol/mol), and 5.9% (41 mmol/mol) were 4.5%, 5.3%, 8.2%, and 30.4%, respectively.

**Table 1:** The effect of different cut-off values of HbA_1c_ on sensitivity, specificity, PVP, PVN, percent of diabetes and area under ROC curves using FPG to diagnose diabetes (cut-off value ≥126 mg/dL).

Cut-off	Sensitivity	Specificity	PVP	PVN	Diabetes	Discriminant
value	%	%	%	%	%	Ability (%)†
≥ 5.5 (37)	91.8	30.1	5.8	98.7	70.9	60.9
≥ 5.6 (38)	83.6	40.3	6.1	98.1	60.8	61.9
≥ 5.7 (39)	80.3	50.2	7.0	98.2	51.2	65.3
≥ 5.8 (40)	78.7	61.3	8.6	98.4	40.5	70.0
≥ 5.9 (41)	75.4	71.1	11.1	98.4	30.4	73.3
≥ 6.0 (42)	72.1	81.6	15.4	98.4	20.8	76.8
≥ 6.1 (43)	70.5	87.9	21.3	98.5	14.7	79.2
≥ 6.2 (44)	67.2	91.7	27.5	98.4	10.9	79.5
≥ 6.3 (45)‡	65.6	94.5	35.7	98.3	8.2	80.0
≥ 6.4 (46)	62.3	96.0	42.2	98.2	6.6	79.1
≥ 6.5 (48)	57.4	97.1	47.9	98.0	5.3	77.3
≥ 7.0 (53)	42.6	99.2	72.1	97.4	2.6	70.9
≥ 8.0 (64)	27.9	99.6	77.3	96.7	1.6	63.7

HbA_1c_ values are % (mmol/mol); PVP: Predictive value positive; PVN: predictive value negative; ROC: Receiver operating characteristics; AUC: Area under ROC curve. **†**Discriminant ability  =  (sensitivity +specificity)/2. **‡**Highest discriminant ability seen for HbA1c of 6.3%.

From a total of 1370 subjects, 61 (4.5%) were diagnosed with diabetes using the criteria of FPG (≥ 126 mg/dL) and 73 (5.3%) were diagnosed having diabetes using the criteria of HbA_1c_ ≥ 6.5% (48 mmol/mol). Thirty five subject were diagnosed with diabetes (2.6%) having both criteria. Thirty eight subjects (2.8%) were diagnosed to have diabetes by HbA_1c_ but not by FPG criteria whereas 26 subjects (1.9%) were diagnosed to have diabetes by FPG but not HbA_1c_ criteria. At a cut-off value of ≥ 6.5% (48 mmol/mol) HbA_1c_ diagnosed 57.4% of subjects to have diabetes from those diagnosed by FPG (≥ 126mg/dL), whereas HbA_1c_ diagnosed 55.8% of subjects to be normal (< 5.7%, 39 mmol/mol) from those diagnosed by FPG (<100 mg/dL). On the other hand, HbA_1c_ diagnosed 628 (45.8%) as having prediabetes (5.7–6.4%, 39–46 mmol/mol) compared to 337 (24.6%) by FPG (100–125 mg/dL), 193 (14.1%) met both criteria.

**Figure 2:**
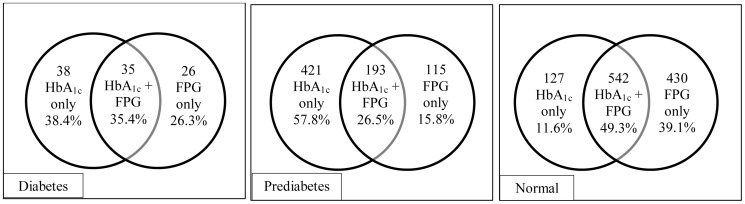
Venn Diagrams for Diabetes, ADA standards. Diabetes diagnosed by HbA_1c_ ≥ 6.5% (48 mmol/mol, n = 73) or FPG ≥ 126 mg/dL (n = 61). Prediabetes diagnosed by HbA_1c_ 5.7–6.4% (39–46 mmol/mol, n = 628) or FPG 100–125 mg/dL (n = 337). Normal diagnosed by HbA_1c_ < 5.7% (39 mmol/mol, n = 669) or FPG <100 mg/dL (n = 972).

The Venn diagrams for diabetes using ADA classification criteria are shown in Figure 2. Only 35.4% of subjects with diabetes meet both FPG and HbA_1c_ criteria whereas 38.4% are diagnosed by HbA_1c_ only and 26.3% by FPG only. In prediabetes Figure 2 shows that only 26.5% have prediabetes with both FPG and HbA1c criteria whereas HbA1c diagnosed 57.8% and FPG diagnosed 15.8%. Approximately 50% of normal subjects are diagnosed by both HbA1c and FPG, however, only 11.6% are diagnosed normal by HbA_1c_ and not by FPG and 39.1% are diagnosed normal by FPG and not by HbA1c.

Table 2 shows that all of the parameters measured (age, systolic BP, Diastolic BP, FPG, HbA1c, TC, TG, LDL and BMI) were significantly higher in subjects with diabetes compared to controls using the criteria of FPG (≥ 126 mg/dL) or HbA_1c_ (≥ 6.5% 48 mmol/mol) to diagnose diabetes except for HDL where the difference between the means was not significant. Other HbA1c cut-off values tested such as 5.5% (37 mmol/mol), 6.0% (42 mmol/mol) and 7.0% (53 mmol/mol) also gave similar differences using t-test to compare means (data not shown).

**Table 2:** The difference between mean values of measured parameters between subjects with diabetes vs. normal subjects according to FPG and HbA_1c_ criteria.

Parameter	Mean ± STD			Mean ± STD		
	Normal by	Diabetes by	p	Normal by	Diabetes by	p
	FPG	FPG	value	HbA1c	HbA1c	value
Age	44.9±7.29	49.3±7.60	.001	44.8±7.26	50.3±7.34	.001
Systolic BP	116±13.3	123±13.7	.001	116±13.3	125±12.8	.001
Diastolic BP	74.2±9.14	77.5±8.87	.006	74.0±9.11	79.2±8.53	.001
FPG mmol/L	5.16±.66	9.52±3.22	.001	5.21±.98	7.80±2.83	.001
HbA_1c_ % (	5.7±.44	7.3±2.02	.001	5.6±.36	7.7±1.63	.001
TC mmol/L	4.58±.96	5.38±1.64	.001	4.60±1.01	4.91±.96	.009
TG mmol/L	1.32±.82	2.38±2.35	.001	1.35±.96	1.81±1.06	.001
HDL mmol/dL	.94±.27	.93±.30	.823	.94±.27	.91±.21	.437
LDL mmol/L	3.05±.79	3.33±.85	.008	3.05±.80	3.24±.72	.047
BMI	30.2±5.57	32.5±5.57	.002	30.2±5.56	32.8±5.62	.001

Diagnosed by FPG (cut-off value ≥126 mg/dL): N for diabetes  =  61, N for normal  =  1309

Diagnosed by HbA_1c_ (cut-off value ≥6.5%), N for diabetes  =  73, N for normal  =  1297

t test was used to compare means of diabetes vs. control.

Pearson correlation coefficients assessed between parameters measured in all recruited subjects were significant (p<0.01) between age and both FPG (r = 0.146) and HbA_1c_ (r = 0.259), FPG and HbA_1c_, TC, TG, LDL and BMI (r = 0.584, 0.242, 0.294, 0.135 and 0.133, respectively). HbA_1c_ correlations with the above parameters were also similar (r =  0.129, 0.124, 0.111 and 0.166 for TC, TG, LDL and BMI, respectively).

Approximately 47% of the subjects were obese (BMI ≥ 30). Mean comparisons by ANOVA of both HbA_1c_ and FPG in obese subjects increase slightly (5 and 8%, respectively) but significantly (p<0.05) compared to normal subjects (BMI < 25). On the other hand, mean BMI values are significantly higher in diabetes compared to normal subjects based on FPG cut-off value of 126 mg/dL (32.5 vs. 29.7 respectively, p<0.001). There is also a similar increase in mean values of HbA_1c_ and FPG in overweight subjects (BMI 25 to < 30) compared to normal subjects (5.7%, 39 mmol/mol vs. 5.5%, 37 mmol/mol, respectively, for HbA_1c_ and 5.29 vs. 5.07 mmol/L, respectively, for FPG). Table 3 shows that diabetic and cardiovascular risk factors were nearly the same whether subjects were diagnosed by HbA1c or FPG or both. The only difference among tested parameters was the TC where the number of diabetic subjects diagnosed by HbA1c or both FPG and HbA1c was statistically higher than those diagnosed by FPG.

**Table 3:** Characteristics of individuals participating in the study according to HbA1c, FPG or both.

Parameter		HbA1c		FPG		HbA1c and FPG	
		Nondiabetic	Diabetic	Nondiabetic	Diabetic	Nondiabetic	Diabetic
**Sex**	Male	450 (34.0%)	25 (34.2%)	443 (33.8%)	25 (41.0%)	432 (34.0%)	14 (40.0%)
	Female	873 (66.0%)	48 (65.8%)	869 (66.2%)	36 (59.0%)	839 (66.0%)	21 (60.0%)
**Diastolic BP (mm Hg)**	≤ 80 mm Hg	120 (94.2%)	65 (89.0%)	1226 (94.0%)	56 (91.8%)	1192 (94.3%)	33 (94.3%)
	> 80 mm Hg	76 (5.8%)	8 (11.0%)	78 (6.0%)	5 (8.2%)	72 (5.7%)	2 (5.7%)
**Systolic BP (mm Hg)**	≤ 120 mm Hg	1252 (95.1%)	65 (89.0%)	1240 (95.1%)	55 (90.2%)	1206 (95.4%)	33 (94.3%)
	> 120 mm Hg	64 (4.9%)	8 (11.0%)	64 (4.9%)	6 (9.8%)	58 (4.6%)	2 (5.7%)
**Family History for Diabetes**	Yes	730 (56.2%)	38 (62.3%)	727 (55.5%)	53 (72.6%)	69 (55.3%)	21 (60.0%)
	No	568 (43.8%)	23 (37.7%)	583 (44.5%)	20 (27.4%)	562 (4 4.7%)	14 (40.0%)
**TC (mmol/L)**	< 5.5 mm Hg	1101 (84.5%)	52 (71.2%)	1109 (84.6%)	40 (65.6%)	1081 (85.1%)	26 (74.3%)
	≥ 5.5 mm Hg	202 (15.5%)	21 (28.8%)	202 (15.4%)	21 (34.4%)	189 (14.9%)	9 (25.7%)
**TG (mmol/L)** †	< 2.0 mmol/L	1112 (85.4%)	53 (72.6%)	1128 (86.1%)	33 (54.1%)	1096 (86.4%)	24 (68.6%)
	≥ 2.0 mmol/L	190 (14.6%)	20 (27.4%)	182 (13.9%)	28 (45.9%)	173 (13.6%)	11 (31.4%)
**HDL (mmol/L)**	< 1.0 mmol/L	829 (63.5%)	50 (68.5%)	839 (63.9%)	38 (62.3%)	811 (63.8%)	25 (71.4%)
	≥ 1.0 mmol/L	475 (36.4%)	23 (31.5%)	473 (36.1%)	23 (37.7%)	460 (36.2%)	10 (28.6%)
**LDL (mmol/L)**	< 3.5 mmol/L	954 (73.7%)	44 (60.3%)	956 (73.3%)	38 (63.3%)	934 (74.0%)	24 (68.6%)
	≥ 3.5 mmol/L	341 (26.3%)	29 (39.7%)	348 (26.7%)	22 (36.7%)	329 (26.0%)	11 (31.4%)
**BMI**	< 25	216 (16.5%)	4 (5.6%)	214 (16.5%)	2 (3.4%)	211 (16.7%)	2 (5.7%)
	25 – < 30	496 (37.9%)	19 (26.8%)	487 (37.5%)	19 (32.8%)	478 (37.9%)	11 (31.4%)
	≥ 30	597 (45.6%)	48 (67.6%)	597 (46.0%)	37 (63.8%)	571 (45.3%)	22 (62.9%)

† p = 0.013 between diabetic subjects diagnosed by HbA1c, FPG, and HbA1c+FPG.

Diabetic [HbA1c ≥ 6.5%, FPG ≥ 126 mg/dL (≥ 7.0 mmol/L)]; Nondiabetic [HbA1c < 6.5%, FPG < 126 mg/dL (< 7.0 mmol/L)].

## Discussion

This study demonstrated the feasibility of using HbA1c in Palestinian Arabs to diagnose diabetes with area under the ROC curve of 0.819. The ideal cut-off value from the ROC curve was approximately 5.9% (equal sensitivity and specificity), however, the optimal cut-off value with the highest discriminant ability was 6.3%. This cut-off value is in agreement with the study on Abu Dhabi Arab population of the United Arab Emirates, UAE, [_ENREF_2525] which reported a cut-off value of 6.4%. At cut-off value of ≥ 6.3%, the sensitivity, specificity, and the discriminant ability, were comparable between this study and that of Abu Dhabi (65.6%, 94.5%, 80% compared to 72.0%, 84.3%%, 78%, respectively). The lower percentage of subjects diagnosed with diabetes by HbA_1c_ between this study and that of the Abu Dhabi study (8.2% vs. 21.0%, respectively) was not surprising since the prevalence of diabetes in the UAE (25% in UEA citizens, and 20% in UAE) is the second highest in the world [26] and subjects at high risk (HbA1c ≥ 6.1%, 43 mmol/mol, and obese with mean BMI 30.4 kg/m^2^) were chosen in the Abu Dhabi study. The slight difference in cut-off values between the two studies could be due to the difference in reference methods used to diagnose diabetes (FPG in this study vs. OGTT in Abu Dhabi study) or the variability within the Arab ethnicity. The study on Arabs living in the United States reported an HbA_1c_ cut-off value of 6.2% [24], but it is not clear from the study if the Arabs were from the same origin. Since true negatives are valued higher than true positives according to the recommendations of the International Expert Committee [3], a cut-off value of ≥ 6.5% (48 mmol/mol) with high specificity (97.1) and a reasonable sensitivity (57.4%) is recommended instead of the cut-off value of 6.3% (45 mmol/mol). At this cut-off value (≥ 6.5%, 48 mmol/mol), HbA_1c_ correctly diagnosed 57.4% as having diabetes and 57.3% as having prediabetes from those diagnosed by FPG. These values are higher than those reported by Pinelli et al. [24] for Arabs in the United States at the recommended cut-off value of ≥ 6.5% (19% for diabetes, 14% for prediabetes). No values were reported for prediabetes in the Abu Dhabi Arab study [25], however, a previous study reported 5% in men and 7% in women having IFG compared to 24.6% in this study [26].

More subjects were diagnosed as having diabetes and prediabetes using HbA_1c_ cut-off value of ≥ 6.5% (48 mmol/mol) for diabetes and 5.7–6.4% (39–46 mmol/mol) for prediabetes (5.3% and 45.8%, respectively) compared to FPG (4.5% and 24.6%, respectively). When comparing FPG (≥ 126 mg/dL, ≥ 7.0 mmol/L) and HbA1c (≥ 6.5%, 48 mmol/L) to diagnose diabetes HbA1c diagnosed 73 subjects compared to 61 subjects by FPG from a total of 1370 subjects 35 subjects where identified by both methods. This indicated a bad agreement between the two methods to recognize the same diabetic subjects. This was also the case in prediabetes where HbA1c diagnosed 628 and FPG 337 subjects from a total of 1370 subjects, only 193 were diagnosed by both methods which indicated that the two methods recognize different populations. Most previous studies reported HbA_1c_ to diagnose less subjects with diabetes compared to FPG or OGTT [4], [27], [28]. This could be due to the delay in analysis that affected FPG due to glycolysis more than HbA_1c_ since samples are transported to a central laboratory [18]. Other studies still reported higher percentages of detecting undiagnosed diabetes by HbA1c ≥ 6.5% (48 mmol/mol) compared to FPG ≥ 126 mg/dl [29]–[31]. Diagnosing higher percentages of prediabetes using HbA1c compared to FPG from this study is consistent with most previously published reports [18], [30], [32].

Correlation between HbA1c ≥ 6.5% and diabetes as diagnosed by FPG was moderate (ĸ = 0.498). This is consistent with recent studies on Korean subjects (ĸ = 0.5) reported by Seo and Lee [33] and Peru subjects (ĸ = 0.41) reported by Miranda et al. [31]. However Cavagnolli et al. [27] and Pinelli at al. [24] reported poor correlation (ĸ = 0.217 and 0.2835, respectively). Not surprisingly, both studies reported low sensitivity for HbA1c ≥ 6.5% (20.9% and 19%, respectively) compared to this study and most other studies that reported sensitivity close to 60% [15], [25], [30], [34], [35]. The above two studies with poor correlation and low sensitivity could be due to the study subjects with mixed ethnicity (Arabs in the United States and Southern Brazilians).

The lack of effect of age, sex and BMI on the diagnostic criteria of HbA1c as compared to FPG is consistent with previous studies [20], [24], [36]. Age-stratified analysis on the feasibility of using HbA_1c_ to diagnose diabetes and prediabetes are consistent with the findings of Penelli et al. [24]. Identifying subjects with diabetes by HbA1c was not affected by age. However, the sensitivity for detecting prediabetes in individuals aged 40–49 years (33.2%) or 50–59 years (37.5%) was significantly higher than those aged 30–39 years (17.1%) (data not shown). There was no difference in the number of subjects with high risk for diabetes and cardiovascular disease diagnosed by HbA1c or FPG except for the parameter TG. This indicates no serious disagreement between the two methods to identify high risk people for diabetes and cardiovascular disease.

The diversity within the Arab ethnic groups requires more studies on using HbA1c to accurately estimate the cut-off values for diagnosing diabetes in different populations. In the Palestinian Arab population raising the cut-off value to 6.5% (48 mmol/mol) increases the percentage of subjects that require preventive measures instead of treatment.
